# Increased Glycated Hemoglobin Levels in Patients With *Helicobacter pylori* Infection Are Associated With the Grading of Chronic Gastritis

**DOI:** 10.3389/fimmu.2020.02121

**Published:** 2020-09-10

**Authors:** Selma Maluf, João Victor Salgado, Dalila Nunes Cysne, Daniel Monte Freire Camelo, Johnny Ramos Nascimento, Bianca Vitória T. Maluf, Luís Douglas Miranda Silva, Marta Regina de Castro Belfort, Lucilene Amorim Silva, Rosane Nassar Meireles Guerra, Natalino Salgado Filho, Flávia Raquel F. Nascimento

**Affiliations:** ^1^Health Science Graduate Program, Federal University of Maranhão, São Luís, Brazil; ^2^Gastroenterology Service of the University Hospital, Federal University of Maranhão, São Luís, Brazil; ^3^Immunophysiology Laboratory, Department of Pathology, Federal University of Maranhão, São Luís, Brazil; ^4^Department of Physiological Sciences, Federal University of Maranhão, São Luís, Brazil; ^5^Department of Pathology, Federal University of Maranhão, São Luís, Brazil; ^6^Department of Medicine I, Federal University of Maranhão, São Luís, Brazil

**Keywords:** *Helicobacter pylori*, glycated hemoglobin, prediabetes, chronic gastritis, cytokines, inflammation

## Abstract

**Background:**

Recent studies have found an association between *Helicobacter pylori* infection and prediabetes. Whether *H. pylori per se* or host factors are involved in the disturbance of glycated hemoglobin needs further investigation. The aim of this study was to determine the association of glycated hemoglobin levels with endoscopic diagnosis and the inflammatory response in *H. pylori* infection.

**Methods:**

A cross-sectional study was carried out in 88 dyspeptic non-diabetic adults who underwent esophagogastroduodenoscopy. The diagnosis of *H. pylori* infection was performed through urease test and histopathological exam. Cases were initially distributed into two groups: control (without *H. pylori* infection, *n* = 22) and HP (patients with *H. pylori* infection, *n* = 66). HbA1c was measured to determine prediabetes status according to the American Diabetes Association criteria, and then the groups were subdivided into non-prediabetic (*n* = 14), prediabetic (*n* = 8), non-prediabetic HP (*n* = 26) and prediabetic HP (*n* = 40) groups. Gastric mucosa was histologically evaluated to determine *H. pylori* density and inflammatory activity according to Sydney System. To investigate the balance of anti-inflammatory and pro-inflammatory cytokines we measured interleukin 10 (anti-inflammatory) and Tumor Necrosis Factor-a (pro-inflammatory) in the plasma or in the gastric mucosa.

**Results:**

Patients with *H. pylori* infection had higher mean HbA1c levels than those without *H. pylori* infection. However, increased HbA1c levels were not associated with *H. pylori*-related factors but with the bacterial density, the intensity of inflammation and the activity of the chronic gastritis. In addition, *H. pylori* infection *per se* did not alter IL-10 and TNF-α neither in the plasma nor in the gastric mucosa, but the bacterial density was negatively correlated with systemic and local IL-10 expression. Although no correlation was found between systemic cytokines and HbA1c levels, local anti-inflammatory cytokine was correlated with HbA1c levels.

**Conclusion:**

Long-term *H. pylori* infection is associated with prediabetes. This association is not related to the presence of *H. pylori per se* but depends on the extent of bacterial colonization and the degree of both local inflammation and activity of the chronic gastritis.

## Introduction

*Helicobacter pylori* infects nearly half of the global population, with a higher prevalence in developing countries (1). The infection is acquired mainly during childhood and may persist in the gastric environment throughout the life of the host, despite the immune and humoral response (2). In Brazil, the prevalence of *H. pylori* infection in adults is between 65 and 82% (3). *H. pylori* infection, a major cause of chronic gastritis, is associated with duodenal and gastric ulcers, lymphoma, and cancer. Furthermore, *H. pylori* has also been linked to extragastric diseases such as type 2 diabetes mellitus (T2DM) (4, 5).

The role of *H. pylori* infection in glucose metabolism and T2DM development has not been fully established. Although it has been reported that patients with chronic *H. pylori* infection have an increased risk of developing T2DM (6), some studies have not found a correlation between this infection and glycemic levels in diabetic patients (7).

The impact of *H. pylori* infection on other organs could be mediated by increased levels of inflammatory markers such as IL-6 and tumor necrosis factor-α (TNF-α), all of which are also involved in the development of insulin resistance and T2DM (5). These cytokines could induce the phosphorylation of serine residues in the insulin receptor substrate, preventing their interaction with insulin receptors, leading to systemic disruption of insulin sensitivity and impaired glucose homeostasis (8, 9).

Few studies have investigated the association between prediabetes and *H. pylori* infection. Yang et al. (10) found that gastric *H. pylori* infection is associated with the risk of diabetes mellitus but not prediabetes. Recent studies have shown a positive association between *H. pylori* infection and prediabetes (11, 12).

The diagnosis of prediabetes is very critical to the preclinical diagnosis of T2DM and further prevention of vascular complications. Glycated hemoglobin (HbA1c), which is used to diagnose prediabetes, is an indicator of long-term glycemic control. Increased HbA1c levels are correlated with an increased risk of T2DM (13). Whether *H. pylori*-related factors or host factors are associated with the disturbance of glycated hemoglobin levels needs investigation.

Therefore, the aim of this study was to determine the association of glycated hemoglobin levels (HbA1c) with endoscopic diagnosis and local and systemic anti- and pro-inflammatory cytokines release in *H. pylori* infection.

## Materials and Methods

### Sample Population

A cross-sectional study was carried out in 88 patients with dyspepsia who underwent esophagogastroduodenoscopy at the Endoscopy Unit of the University Hospital, Federal University of Maranhão (UFMA), during a period of 4 months. The patients eligible for the study were selected through systematic random sampling according to the demands of the Endoscopy Service. Patients of both genders who were ≥18 years of age and who did not have a history of diabetes were included. The following patients were not included in the study: pregnant women, patients with chronic consumptive disease (cancer and AIDS), gastric surgery patients, chronic renal failure patients on hemodialysis, patients with recent blood transfusion, patients previously treated for *H. pylori* eradication, and patients who had been medicated with H2 receptor antagonists or proton pump inhibitors within 2 weeks or antibiotics within 4 weeks prior to testing.

The patients included completed a sociodemographic survey to collect information on educational level, lifestyle, medical history, and medication use and for dyspepsia evaluation. The body mass index (BMI) was also calculated, and the patients were classified as eutrophic (BMI values <25 kg/m^2^) and overweight/obese (BMI values ≥25 kg/m). Any participant who self-declared as a regular smoker, regardless of the quantity of cigarettes smoked, was considered a smoker. The National Committee for Research Ethics (CONEP) approved this study according to CAAEE 07989412.0.0000.5086. Those patients who accepted the invitation to participate in the study signed an informed consent form.

### Diagnosis of *H. pylori* Infection

*Helicobacter pylori* infection was determined using the rapid urease test (RUT) and a histopathological exam. Gastric mucosa samples were collected during endoscopic biopsies. For the RUT, two specimens of gastric mucosa (antrum and body) were collected using autoclavable biopsy forceps and placed in a sterile tube containing urea and a pH indicator (phenol red) (Uretest Promedical, Brazil). The sample was kept at room temperature and after 2 h was read. A color change from dark yellow to red indicated positivity for *H. pylori* infection. For the histopathological exam, four tissue fragments (two fragments each from the body and antrum) were collected using sterile biopsy forceps. The samples were placed in a single vial containing 10% formaldehyde, which was properly labeled and submitted to the Department of Pathology of the HUUFMA. Giemsa staining techniques were used to facilitate bacterial visualization. Identification of *H. pylori* in any test was considered a positive result in accordance with the IVth Brazilian Consensus Conference on *H. pylori* infection (14).

### Endoscopic Diagnosis

A member of the research team performed all endoscopies. An Olympus Exera II system (Olympus Corporation, Tokyo, Japan) was used for digestive endoscopy. Before the esophagogastroduodenoscopy exams, dimethicone drops and lidocaine spray (10% Xylestesin; Cristália, Brazil) were applied to the oropharynx. Sedation was performed using intravenous midazolam (Dormonid Roche, Brazil). During endoscopy, the esophagus, stomach, and duodenum were evaluated, and the endoscopic findings were reported. Diagnoses of chronic gastritis and duodenitis were made based on the Sydney System (15). A peptic ulcer lesion was described as an area of mucosal continuity with a perceived depth of more than 0.5 cm.

### Morphological Analysis of the Gastric Mucosa

The gastric mucosa obtained from the endoscopic biopsy were routinely prepared with paraffin, and sections were cut (5-μm thick) perpendicular to the surface and were stained with hematoxylin and eosin. Two experienced pathologists without knowledge of other data performed the microscopic examinations. Histological analysis evaluated morphological variables according to Updated Sydney System (16): inflammation, activity and density of *H. pylori*. A three-grade system of mild, moderate and severe was applicated to morphological variables according the standard grading scale of the System and each successive grade represents an increment in severity of approximately one-third. Inflammation refers to the presence of mononuclear inflammatory infiltrate in lamina própria and categorizes the gastritis as chronic. We adopt HP 0/1 for mild degree of inflammation; HP 2/3 for moderate and severe degree of inflammation. Activity in the context of chronic gastritis refers to the presence of polymorphonuclear leukocytes alongside the mononuclear inflammatory infiltrate. Activity can be particularly related to the presence of *H. pylori* (17). The neutrophil activity was graded as mild when less than one-third of the epithelial grooves and the surface were infiltrated by neutrophils; it was considered to be of moderate intensity when neutrophil infiltration occurred in one to two-thirds of the surface and severe when more than two-thirds of the surface of the epithelium showed neutrophil infiltration. We use HP 0/1 to grade neutrophil activity as mild; HP 2/3, for moderate and severe degree of activity. The density of *Helicobacter-like* organisms present is grading in mild colonization when individual organisms, or small groups, covering less than one-third of the mucosal surface (1^+^/3^+^). Severe colonization is the presence of large groups of organisms on the surface and upper pits of more than two-thirds of the mucosal surface examined (3^+^/3^+^). Moderate colonization is between these two (2^+^/3^+^). We classify in HP dens1, the mild colonization; HP dens2, the moderate colonization and HP dens3, severe colonization.

### Evaluation of Cytokines in Plasma by CBA

For the evaluation of cytokines, one sample of blood (5 mL) was collected from each patient and sent to the Immunophysiology Laboratory, UFMA. The concentrations of the cytokines IL-2, IL-4, IL-6, IL-10, IL-17, IFN-γ, and TNF-α were determined using a cytometric bead array (CBA) human Th1/Th2/Th17 cytokine kit (Becton Dickinson Biosciences, San Jose, CA, United States). For this study only the data of IL-10 and TNF-a were used. The plasma samples were analyzed in a FACSCalibur flow cytometer (Becton Dickinson Biosciences, San Jose, CA, United States) previously calibrated with “setup beads” incubated with fluorescein isothiocyanate (FITC) or phycoerythrin (PE) according to the manufacturer’s recommendation. A standard curve was generated for each cytokine. The results were analyzed in FCAP Array Software (Becton Dickinson Biosciences, San Jose, CA, United States), and the values were expressed as pg/mL for each cytokine. The limit of detection for each cytokine is defined as the corresponding concentration at two standard deviations above the median fluorescence of 30 replicates of negative control (0 pg/mL).

### Evaluation of Cytokines in Gastric Mucosa by Immunochemistry

Tissue microarray blocks (TMAs) were assembled and the 3 μm thick histological sections were placed on silanized slides (StarFrost^®^ Advanced Adhesive, Knittel, Germany) and taken to an oven at 65°C for 1 h. The gastric tissue was deparaffinized with xylol and dehydrated in alcohol. Endogenous peroxidase enzyme blockade was performed with H_2_O_2_ (hydrogen peroxide) solution (Merck KgaA, Darmstadt, Germany) at 3%. Antigenic recovery was performed by heating the tissue for 40 min at a temperature between 97 and 100°C. Then, the antibody diluted in PBS-Tween 20/BSA 1% was applied over gastric tissue and incubated for 40 min. Rabbit polyclonal anti-IL-10 antibody (Biorbyt, Cambridge, United Kingdom) and anti-TNF-α antibody (Scytek, Utah, United States) were used. The slides were washed with PBS-Tween 20 pH 7.4 solution and incubated with the chromogen 3.3′-Diaminobenzidine (DAB, Sigma, St Louis, MO, United States, D5637-1G) diluted in DAB Substrate, for 5 min, at room temperature. Counterstaining was done with Hematoxylin. The cuts were dehydrated in alcohol and xylol solutions and subsequently assembled. The positive control for TNF-α was performed with lymph node sections. The positive control for IL-10 was performed with kidney sections. The expression of IL-10 and TNF-α was evaluated in epithelial cells and the lamina propria of the gastric mucosa by using an ECLIPSE E800 electronic microscope (Nikon Instruments Europe, Amsterdam, Netherlands) with a 20× objective on the EGFP filter (Emission Green Fluorescent Protein). The counting of the marked cells in five fields of the histological section was performed. The fields were determined as “upper left field,” “upper right field,” “lower left field,” “lower right field,” and “center.” After counting, the sum of the five fields was made to obtain an estimate of the cells marked for IL-10 or TNF-α, per patient slide.

### Statistical Analysis

The absolute and relative frequencies are used to describe the qualitative variables. The mean and standard deviation or median and interquartile range are used to the describe quantitative variables according to the Shapiro–Wilk normality test.

To evaluate the association between the quantitative variables and the presence of *H. pylori* infection, the Mann–Whitney test or Student’s *t*-test for independent samples was used. The Kruskal–Wallis test followed by Dunnett’s test or ANOVA followed by Bonferroni’s test were used to verify the differences in the quantitative variables between the study groups.

Qualitative variables were assessed by Fisher’s exact and Chi-square tests for the presence of *H. pylori* and for the analysis of differences between the study groups. For the correlation analyses, the Spearman test was used. Differences were considered significant when the *p*-value ≤ 0.05. The data were tabulated and analyzed using the data and statistical analysis software STATA^®^ version 14.0 and GraphPad Prism 6.0 software.

## Results

### Evaluation of Sociodemographic Parameters

There were no significant differences regarding age, sex, BMI, gastric symptoms, smoking habits, or alcohol consumption. The majority of the study population consisted of women with an average age of approximately 40 years with BMI less than 25 (Table 1).

**TABLE 1:** General characteristics of patients according to the presence of infection.

Characteristics	*H. pylori*				*p*-Valor
	Negative		Positive		
	n	%	N	%	
Age (years)					
Mean ± SD	39.5 ± 12.8	41.2 ± 11.9	0.9		
Sex					
Male	5	22.7	24	36.3	0.3
Female	17	77.2	42	63.6	
HbA1c (%)					
Mean ± SD	5.5 ± 0.3	5.7 ± 0.4	**0.0**		
BMI (kg/m^2^)					
Median (interquartile range)	23.5 (25.9–22.3)	24.4 (27.6–22.8)	0.2		
HbA1c (%)					
<5,7	16	68.1	30	45.4	0.0
≥ 5,7	7	31.8	36	54.5	
BMI (kg/m^2^)					
<25	14	63.6	37	56.0	0.5
≥25	8	36.3	29	43.9	
Symptoms					
Epigastric pain	12	54.5	31	46.9	0.4
Fullness	1	4.5	11	16.6	
Epigastric pain + fullness + satiety	9	40.9	24	36.3	
Endoscopy					
Normal	5	22.7	13	19.7	0.0
Pangastritis	7	31.8	38	57.5	
Peptic ulcer	10	45.4	15	22.7	
Smoking habits					
Yes	2	9.0	4	6.1	0.6
No	20	90.9	61	93.8	
Alcoholism					
Yes	4	19.0	55	85.9	0.7
No	17	80.9	9	14.0	

### Prevalence of *H. pylori* Infection, Symptoms, and Endoscopic Findings

Approximately 75% of patients were infected with *H. pylori*. Most of them were women aged 18–54 years. Half of the patients complained of epigastric pain. Enanthematous pangastritis was the most frequent endoscopic finding in patients infected with *H. pylori* (Table 1).

### *Helicobacter pylori* Infection Was Associated to Increased HbA1c Levels

**FIGURE 1:**
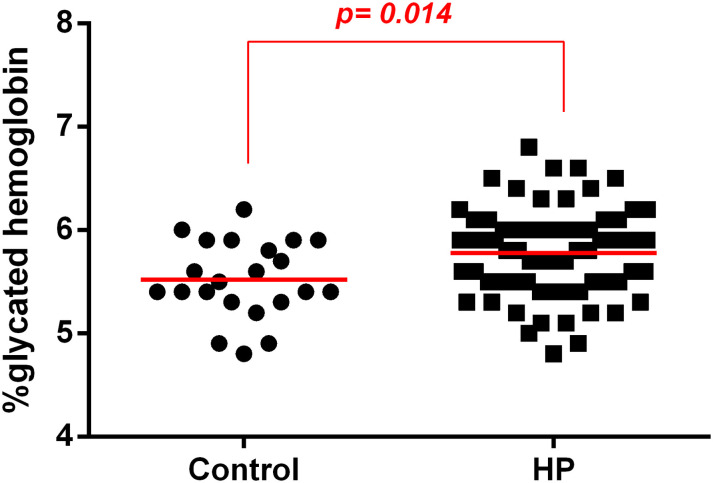
Association between *H. pylori* infection (HP) and glycated hemoglobin (HbA1c) levels in patients without infection (control) or with *H. pylori* infection (HP). HbA1c levels were evaluated in the plasma of patients infected with *H. pylori* (HP, *n* = 66) or non-infected (Control, *n* = 22) using a method certified by the National Glycohemoglobin Standardization Program (NGSP). The line represents mean of the group. *p*-value was determined by unpaired two-tailed *t*-test. Level of significance, *p* < 0.05.

Patients with *H. pylori* infection had higher mean HbA1c levels than patients without *H. pylori* infection (Figure 1).

### There Was an Association Between Increased *H. pylori* Density and the Increase in HbA1c Levels

**FIGURE 2:**
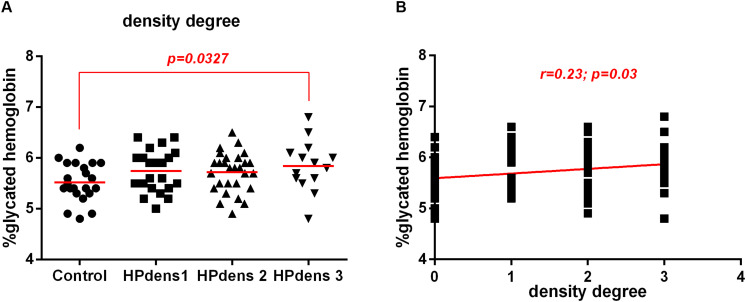
Association between *H. pylori* infection (HP) and glycated hemoglobin (HbA1c) levels in patients without infection (Control) or with *H. pylori* infection (HP) according to degree bacterial density of the gastric mucosa. Bacterial density of the gastric mucosa were evaluated according Sydney System histological classification of patients infected with *H. pylori* (HP, *n* = 66) or non-infected (Control, *n* = 22). **(A)** Degree of bacterial density of the non-infected (Control, *n* = 22), *H. pylori* positive with density 1 (HP dens1, *n* = 23), *H. pylori* positive with density 2 (HP dens2, *n* = 29), *H.* pylori *positive* with density 3 (HP dens3, *n* = 14). The line represents mean of the group. *p*-value was determined by unpaired two-tailed *t*-test. **(B)** Correlation between HbA1c levels and bacterial density. Correlations were determined by Spearman’s correlation test. Level of significance, *p* < 0.05.

The mean HbA1c levels were higher in the *H. pylori*-infected group with a higher bacterial density 3^+^/3^+^ (HPdens3) than in the control group. There was no significant difference between controls and low-density infected patients (HPdens1) as well as between controls and infected patients with moderate bacterial density (HPdens2). There were also no significant differences between the infected groups HPdens1 and HPdens2, HPdens2 and HPdens3, and HPdens1 and HPdens3 (Figure 2A). However, bacterial density was positively correlated with HbA1c levels (Figure 2B).

### Endoscopic Diagnosis Was Not Associated With High Levels of HbA1c, but Rather the *H. pylori* Related-Factors

**FIGURE 3:**
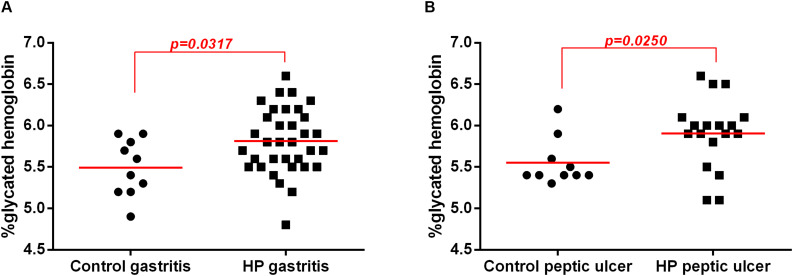
Association between *H. pylori* infection (HP) with glycated hemoglobin (HbA1c) levels in patients without infection (Control) or with *H. pylori* infection (HP) according endoscopic diagnosis. Endoscopic diagnosis were evaluated according endoscopic classification of the Sydney System of patients infected with *H. pylori* (HP, *n* = 52) or non-infected (Control, *n* = 20. **(A)** Enanthematous pangastritis endoscopic diagnosis of *H. pylori*-infected (HP gastritis, *n* = 34) or non-infected (Control gastritis, *n* = 10). **(B)** Peptic ulcer endoscopic diagnosis of *H. pylori*-infected (HP peptic ulcer, *n* = 18) or non-infected (Control peptic ulcer, *n* = 10). The line represents median of the group. *p*-value was determined by non-parametric Mann–Whitney test. Level of significance, *p* < 0.05.

Among patients with endoscopic diagnoses of pangastritis and among patients with peptic ulcers, HbA1c levels were higher in patients with *H. pylori* infection than in those without infection (Figures 3A,B). When we compared patients presenting peptic ulcer, but without *H. pylori* infection with patients presenting gastritis, but with *H. pylori* infection we observed that HbA1c levels were higher in patients with *H. pylori* infection and gastritis diagnosis (Supplementary Figure S1). These data suggest that the endoscopic diagnosis does not appear to be associated with HbA1c levels, but rather the *H. pylori* related-factors.

### There Was an Association Between Increased HbA1c Levels With Severe Degree of Inflammation and Activity of Chronic Gastritis

**FIGURE 4:**
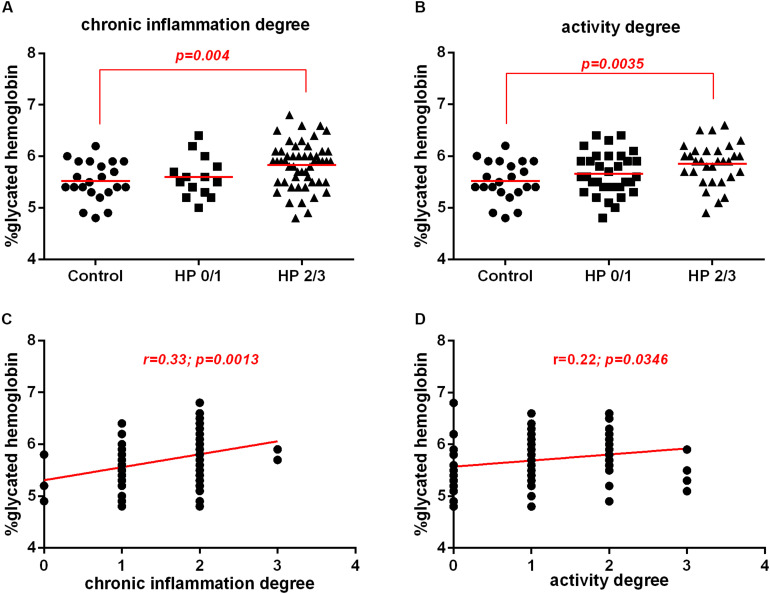
Association between *H. pylori* infection (HP) with glycated hemoglobin (HbA1c) levels in patients without infection (Control) or with *H. pylori* infection (HP) according morphological analysis of the gastric mucosa. Histological analysis was evaluated by Sydney System histological classification of patients infected with *H. pylori* (HP, *n* = 66) or non-infected (Control, *n* = 22). **(A)** Degree of chronic inflammation of non-infected with normal mucosa, or mild chronic inflammation (Control, *n* = 22), *H. pylori-*infected with mild chronic inflammation (HP0/1, *n* = 14), *H. pylori* infection patients with moderate and severe gastric mucosa chronic inflammation (HP2/3, *n* = 52). **(B)** Degree of neutrophil activity of non-infected with no activity or mild activity (Control, *n* = 22) or *H. pylori* infected with mild activity (HP0/1, *n* = 35), *H. pylori* infected with moderate and severe gastric mucosa activity (HP2/3, *n* = 31). The line represents the mean of the group. *p*-value was determined by unpaired two-tailed *t*-test **(C)** Correlation between HbA1c levels with chronic inflammation of the gastric mucosa. **(D)** Correlation between HbA1c levels with of neutrophil activity of the gastric mucosa. Correlations were determined by Spearman’s correlation test. Level of significance, *p* < 0.05.

There was an increase in HbA1c levels in patients infected with *H. pylori* with moderate and severe chronic inflammation of the gastric mucosa compared to those in the control group (no infection with normal mucosa or mild inflammation). However, there was no change in the HbA1c levels between the control group and the HP group with mild inflammation (Figure 4A).

Similarly, HbA1c levels were increased in *H. pylori*-infected patients with moderate to high gastric mucosa activity compared to those in the control group. There was no change in HbA1c levels between the control group and the HP group with mild activity (Figure 4B). Furthermore, chronic inflammation and neutrophil activity were positively correlated with the HbA1c levels (Figures 4C,D). These data suggest that the intensity of the neutrophil activity and the inflammation of the chronic gastritis may be predisposing factors for prediabetes.

### The Presence of *H. pylori* Infection Did Not Alter Systemic Levels of IL-10, TNF-α or Alter the Expression of the Cytokines TNF-α and IL-10 in Gastric Mucosa

**FIGURE 5:**
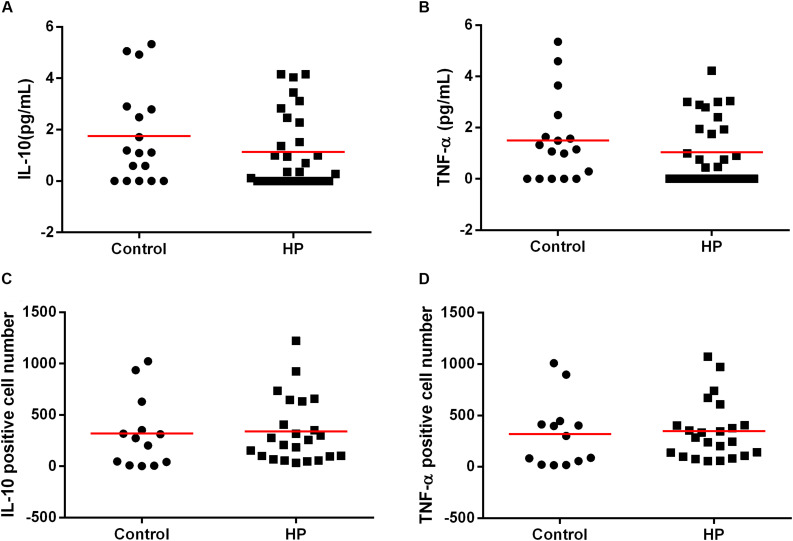
Association between *H. pylori* infection (HP) with IL-10 and TNF-α cytokines in plasma and in gastric mucosa of patients without *H. pylori* infection (Control) and with *H. pylori* infection (HP). Cytokines concentration in plasma were measure by CBA of patients infected with *H. pylori* (HP, *n* = 30) or non-infected (Control, *n* = 17). Concentration of IL-10 **(A)** and TNF-α **(B)** in plasma of patients control and HP. The line represents mean of the group. *p*-value was determined by unpaired two-tailed *t*-test. Expression of cytokines in the gastric mucosa was evaluated by immunohistochemistry of the infected patients (HP, *n* = 24) or non-infected group (Control, *n* = 13). Expression of IL-10 **(C)** and TNF-α **(D)** in gastric mucosa of patients control and HP. The line represents median of the group. *p*-value was determined by non-parametric Mann–Whitney U test. Level of significance, *p* < 0.05.

Considering that the inflammation of the chronic gastritis may be predisposing factors for prediabetes, we evaluated two antagonistic cytokines, IL-10 (anti-inflammatory) and TNF-a (pro-inflammatory) in the plasma and in the gastric mucosal and tested if they could be associated with the presence of the bacterium by itself. However, there was no difference in IL-10 or TNF-α levels in the plasma of *H. pylori-*infected patients compared to those in non-infected patients (Figures 5A,B). Similarly, there was no difference in the IL-10 or TNF-α expression in the gastric mucosa of *H. pylori*-infected patients compared to those non-infected patients (Figures 5C,D).

### Bacterial Density and the Activity of the Chronic Gastritis Were Associated With IL-10 Levels in Plasma

Since we observed that the bacterial density as well as the intensity of gastric mucosa activity and the chronic inflammatory process could be predisposing factors for prediabetes in *H. pylori*-infected patients, we investigated whether the cytokines were associated with inflammation of the gastric mucosa and the bacterial load. Our results showed a decrease in the expression of IL-10 in plasma and in the gastric mucosa in *H. pylori*-infected patients with increased bacterial density and a higher degree of neutrophil activity.

**FIGURE 6:**
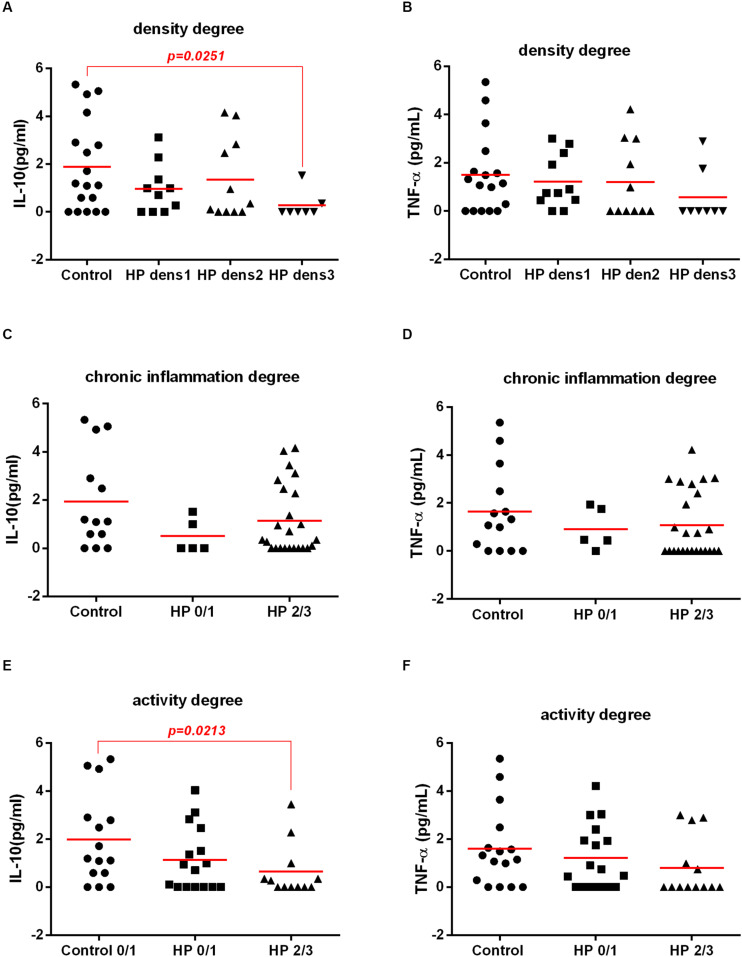
Association between *H. pylori* infection (HP) with IL-10 and TNF-α cytokines in plasma of patients without *H. pylori* infection (Control) and with *H. pylori* infection (HP) according morphological analysis of gastric mucosa. Cytokines concentration in plasma were measure by CBA and histological analysis of the gastric mucosa was evaluated by Sydney System histological classification of patients infected with *H. pylori* (HP, *n* = 30) or non-infected (Control, *n* = 18). Concentration in plasma of IL-10 **(A)** and TNF-α **(B)** according degree of bacterial density of non-infected (Control, *n* = 18), infected-*H. pylori* with grade 1 density (HP dens1, *n* = 10), infected-*H. pylori* with grade 2 density (HP dens2, *n* = 11), infected-*H. pylori* with grade 3 density (HP dens3, *n* = 7). Concentration in plasma of IL-10 **(C)** and TNF-α **(D)** according degree of chronic inflammation of non-infected with normal mucosa, or mild chronic inflammation (Control, *n* = 14), *H. pylori-*infected with mild chronic inflammation (HP0/1, *n* = 5), *H. pylori* infection patients with moderate and severe gastric mucosa chronic inflammation (HP2/3, *n* = 25). Concentration in plasma of IL-10 **(E)** and TNF-α **(F)** according neutrophil activity degree of non-infected with no activity or mild activity (Control, *n* = 12) or *H. pylori* infected with mild activity (HP0/1, *n* = 16), *H. pylori* infected with moderate and severe gastric mucosa activity (HP2/3, *n* = 12). The line represents median of the group. *p-*value was determined by non-parametric Mann–Whitney U test. Level of significance, *p* < 0.05.

In the plasma, IL-10 levels decreased in the group HPdens3 when compared with control, but there was no significant difference between HPdens1 and the control or HPdens2 and the control. Additionally, no difference was found in plasmatic IL-10 levels when comparing the infected groups HPdens1 with HPdens2, HPdens2 with HPdens3 and HPdens3 with HPdens1 (Figure 6A). Furthermore, IL-10 levels in plasma was correlated negatively with the bacterial density (Supplementary Figure S2).

There was no significant association between plasmatic IL-10 levels and the degree of chronic inflammation (Figure 6C). However when the plasmatic expression of the cytokine IL-10 and the activity of the chronic gastritis were analyzed, a significant decrease in IL-10 was observed in the infected groups with moderate and intense gastric mucosa activity (HP2/3) compared with that in the non-infected group with mild activity (Control). However, there was no significant difference between the HP1-infected group and the control group. In addition, there was no significant difference in plasmatic IL-10 levels between the HP1 and HP 2/3 groups (Figure 6E). There was no significant association between the TNF-α levels in plasma with the results of the morphological analysis (Figures 6B,D,F) or the bacterial density (Supplementary Figure S2).

### Bacterial Density and the Activity and Chronic Inflammation of the Gastric Mucosa Were Associated With IL-10 Levels in Gastric Mucosa by Immunohistochemistry

**FIGURE 7:**
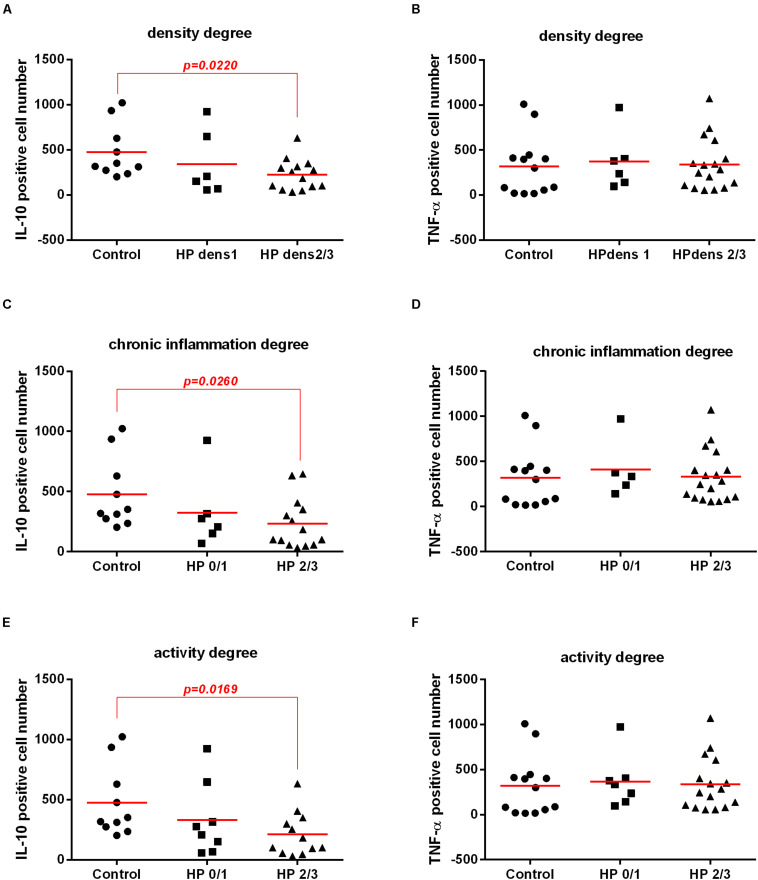
Association between *H. pylori* infection (HP) with IL-10 and TNF-α cytokines expression in gastric mucosa of patients without *H. pylori* infection (Control) and with *H. pylori* infection (HP) according morphological analysis of gastric mucosa. Expression of IL-10 and TNF-α in gastric mucosa were evaluated by immunochemistry and histological analysis of the gastric mucosa was evaluated by Sydney System histological classification of patients infected with *H. pylori* (HP, *n* = 20) or non-infected (Control, *n* = 10). Expression in gastric mucosa of IL-10 **(A)** and TNF-α **(B)** according degree of bacterial density of the non-infected (Control, *n* = 10), infected-*H. pylori* with grade 1 density (HP1, *n* = 6), infected-*H. pylori* with grade 2 and grade 3 density (HP2/3, *n* = 14). Expression in gastric mucosa of IL-10 **(C)** and TNF-α **(D)** according degree of chronic inflammation of non-infected with normal mucosa, or mild chronic inflammation (Control, *n* = 10), *H. pylori-*infected with mild chronic inflammation (HP0/1, *n* = 8), *H. pylori* infection patients with moderate and severe gastric mucosa chronic inflammation (HP2/3, *n* = 12). Expression in gastric mucosa of IL-10 **(E)** and TNF-α **(F)** according degree of gastric mucosa activity of non-infected with no activity or mild activity (Control, *n* = 10) or *H. pylori* infected with mild activity (HP0/1, *n* = 8), *H. pylori-*infected with moderate and severe gastric mucosa activity (HP 2/3, *n* = 12). The line represents median of the group. *p*-value was determined by non-parametric Mann–Whitney U tests. Level of significance, *p* < 0.05.

IL-10 expression was lower in HPdens2/3 than in the control, but there was no significant difference between HPdens1 with control; in addition, there was no difference between HPdens1 with HPdens2/3 (Figure 7A). According to the degree of chronic inflammation, there was decreased IL-10 expression in *H. pylori*-infected with moderate/intense chronic inflammation (HP 2/3) compared with that in the control. However, there was no significant difference between *H. pylori*-infected with low chronic inflammation (HP 0/1) and the control. In addition, there was no significant difference between infected groups HP 0/1 and HP2/3 (Figure 7C). According to activity of the chronic gastritis, there was decreased IL-10 expression in *H. pylori*-infected with moderate/intense activity (HP2/3) compared with that in the control (non-infected, with no activity or low activity). However, there was no significant difference between *H. pylori*-infected with low activity (HP 0/1) and the control. In addition, there was no significant difference between infected groups HP 0/1 and HP2/3 (Figure 7E). There was no significant difference in TNF-α level in the gastric mucosa of *H. pylori*-infected patients according to morphological analysis (Figures 7B,D,F).

### The Expression of IL-10 and TNF-α in the Gastric Mucosa Were Positively Correlated With HbA1c Levels

**FIGURE 8:**
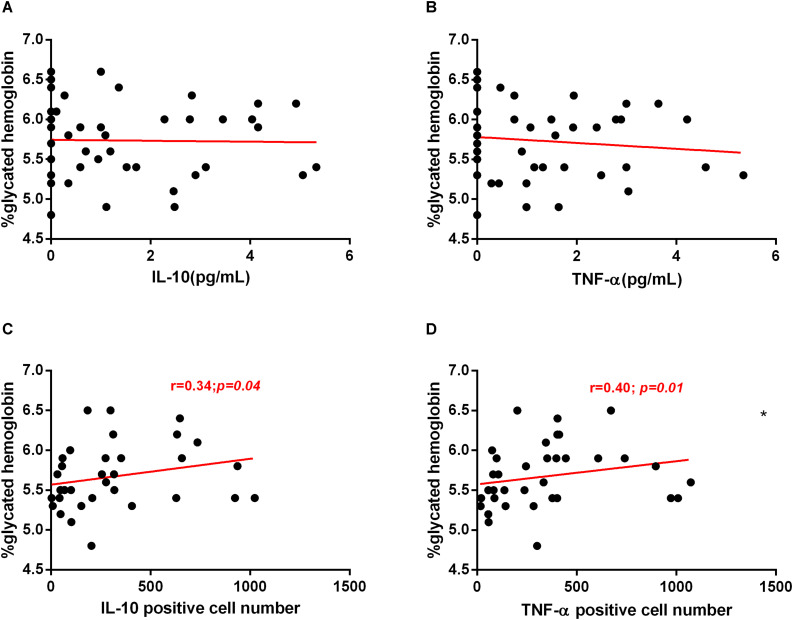
Correlation between HbA1c levels with IL-10 and TNF-α cytokines, in plasma and in gastric mucosa. Cytokines concentration in plasma were measure by CBA. Correlation between HbA1c with IL-10 **(A)** and TNF-α **(B)** plasma concentration of 47 patients. Expression of cytokines in the gastric mucosa was assessed by immunohistochemistry. Correlation between HbA1c with expression of IL-10 **(C)** and TNF-α **(D)** in gastric mucosa of 37 patients. Correlations were determined by Spearman’s correlation tests Level of significance, *p* < 0.05.

There was no correlation between the plasma levels of IL-10, TNF-α with the HbA1c levels (Figures 8A,B). However, the expression of IL-10 and TNF-α in the gastric mucosa was positively correlated with the HbA1c levels (Figures 8C,D), suggesting that inflammatory and anti-inflammatory markers released in the gastric mucosa during infection by *H. pylori* may be involved in the disturbance of glucose metabolism.

### There Was No Correlation Between the Levels of Plasma Cytokines and Gastric Mucosa Cytokines

**FIGURE 9:**
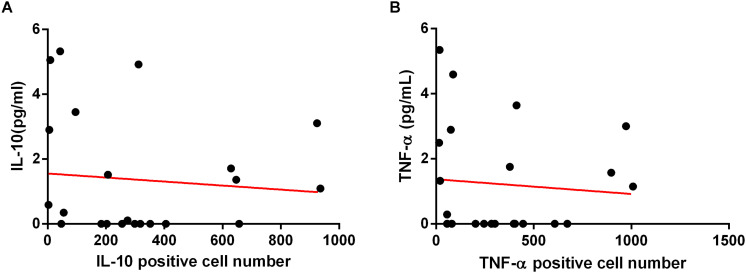
Correlation between cytokines IL-10 with TNF-α, in plasma and in gastric mucosa. Cytokines concentration in plasma were measure by CBA and expression of cytokines in the gastric mucosa was evaluated by immunohistochemistry. **(A)** Correlation between IL-10 plasma concentration with IL-10 expression in gastric mucosa of 24 patients. **(B)** Correlation between TNF-α concentration in plasma with TNF-α expression in gastric mucosa. Correlations were determined by Spearman’s correlation tests. Level of significance, *p* < 0.05.

There was no correlation between the plasma levels of IL-10 with expression of IL-10 in the gastric mucosa (Figure 9A). Similarly, there was no correlation between the plasma levels of TNF-α with the expression of TNF-α in the gastric mucosa (Figure 9B).

## Discussion

The present study showed that patients infected with *H. pylori* had higher mean HbA1c levels than non-infected patients, suggesting a positive association between *H. pylori* infection and prediabetes. However, this association is not related to the presence of *H. pylori* by itself but depends on the density of bacterial colonization and the degree of both inflammation and the activity of the chronic gastritis.

The association of *H. pylori* with prediabetes and diabetes is still controversial. According to a systematic review and meta-analysis, *H. pylori* infection was associated with higher HbA1c levels in patients with type I diabetes but not with T2DM (18). In contrast, in other studies of patients with T2DM, a positive association between *H. pylori* infection and suboptimal glycemic control was shown (19, 20). A positive association was previously noted between *H. pylori* infection and prediabetes (11, 12). Draz et al. (11) assessed the association between HP and prediabetes in adults using the oral glucose tolerance test (OGTT) for prediabetes diagnosis and histopathological examination for *H. pylori* diagnosis. In the present study, we used HbA1c to infer prediabetes status. The use of HbA1c has several advantages over the use of fasting plasma glucose and OGTT, which include increased convenience (fasting is not required), greater preanalytical stability and reduced day-to-day variation due to stress and illness (13). We employed the RUT and histopathological examination for *H. pylori* diagnosis. The RUT is a highly accurate invasive test for the diagnosis of *H. pylori* infection (21). By collecting body and antrum tissue fragments, as we did in this study, we reduced the probability of false negative results (22). Histological examination is considered the gold standard method for detecting *H. pylori* infection and allows morphological analysis of the gastric mucosa (23).

Several hypotheses exist regarding the role of gastric *H. pylori* infection in glucose regulation and type 2 diabetes development. *H. pylori* titers were shown to be independent predictors of hyperglycemia and abnormal pancreatic β-cell function, despite their relatively low adiposity (24). In addition, *H. pylori* infection induced insulin resistance in hepatocytes by activating the c-Jun/miR-203/SOCS3 signaling pathway (25). Furthermore, *H. pylori* infection may induce local and systemic increases in proinflammatory cytokines by inducing structural changes in insulin receptors and the inhibition of insulin action (9).

This study was designed to investigate whether *H. pylori per se* or host-related factors are involved in the disturbance of glycated hemoglobin. We observed that *H. pylori-*infected patients had higher mean HbA1c levels than those not infected with *H. pylori.* It was interesting to note that when the groups were stratified into HP-infected and non-infected groups and into prediabetic and non-prediabetic groups, it was observed that even in the patients considered non-prediabetic but who were infected with HP, HbA1c levels were higher than those in patients considered non-prediabetic but who were not infected with HP (Supplementary Figure S3), suggesting that HP is an important agent related to the increase in HbA1c.

Then, we compared the endoscopic diagnosis results with the HbA1c levels and observed that among patients diagnosed with pangastritis, HbA1c levels were significantly higher in those with *H. pylori* infection than in those without infection. Similarly, among patients with peptic ulcers, HbA1c levels were significantly higher in those with *H. pylori* infection than in those without infection. Moreover, when we compared a group of patients with endoscopic diagnosis of pangastritis associated with *H. pylori* infection with another group of patients with endoscopic diagnosis of peptic ulcer who were not infected, the HbA1c levels were significantly higher in the group with *H. pylori* infection than in the group without infection. These data have allowed us to initially assume that *H. pylori-*related factors could induce disturbances in HbA1c levels.

Interestingly, when we histologically evaluated the gastric mucosa, a significant increase in HbA1c levels was noted in the group of infected patients with moderate and intense activity and chronic inflammation of the gastric mucosa when compared to that in the controls. However, there was no change in HbA1c levels between the control group and the infected group with mild inflammation and low neutrophil activity. These data suggest that the degree of chronic inflammation and the degree of the activity of the gastric mucosa can influence HbA1c levels. Furthermore, we observed increased HbA1c levels in *H. pylori*-infected patients with increased bacterial density. Studies show that increased bacterial density is associated with a more intense active chronic inflammatory process (26). Thus, we support the hypothesis that the severity of the inflammatory process in the gastric mucosa is a risk factor for prediabetes.

The cross-sectional design of this study is a limitation because only allows us to establish associations, not being able to define a cause-effect relationship. However, epidemiological studies showed that *H. pylori* infection is acquired in childhood (27). In developing countries the rate of acquisition of the pathogen can reach up to 50% of children at the age of five (28), similar to the prevalence rate in adults (1). Natural clearance of *H. pylori* infection is difficult to determine and infection can perpetuate throughout the life of the host (2). Therefore, it is possible to believe that *H. pylori* infection occurred long time before T2DM. Jeon et al. (29) using a prospective cohort demonstrated that individuals seropositive for *H. pylori* whose diabetic status was not known at the initiation of the study were two times more likely to develop diabetes than those who were seronegative, even after adjusting for age, sex, education, and covariates such as smoking, BMI, blood pressure, and lipids. Chen and Blaser (30) showed that *H. pylori* seropositivity, and *H. pylori* cagA positivity in particular, was associated with higher mean HbA1c levels, an association that persisted after excluding individuals with a history of diabetes mellitus and controlling for potential confounders. Study performed by Hsieh et al. (31) observed that long-term *H. pylori* infection was significantly associated with high levels of HbA1c, decreased insulin secretion, and a higher prevalence of T2DM in Taiwanese patients.

The pathogenesis of T2DM has been associated with a subclinical chronic inflammation and activation of the immune system. *H. pylori* infection will invariably induce chronic low-grade inflammation with positive regulation of pro-inflammatory cytokines and mediators, such as C-reactive protein, tumor necrosis factor and interleukine-6, which may affect peripheral insulin action and secretion of pancreatic β cells (32, 33).

Type 2 diabetes mellitus is characterized by a metabolic disorder between increased circulating levels of pro-inflammatory cytokines like interleukin- IL-1, IL-6, tumor necrosis factor-(TNF-)α, transforming growth factor-β (TGF-β) and decreased anti-inflammatory cytokines like IL-4, IL-10, IL-13 (34), which are involved with peripheral insulin resistance (8) and beta cell apoptosis in T2DM (34).

In this study, there was no significant differences in the plasma levels between anti-inflammatory (IL-10), and pro-inflammatory cytokine (TNF-α) between groups with and without *H. pylori* infection. There was also no association between plasma levels of interleukins IL-10 and TNF-α with HbA1c levels. Similar results described by Yang et al. (35) showed that chronic *H. pylori* infection affects glucose metabolism, but plasma levels of systemic mediators were not involved in these effects. We showed that *H. pylori* infection might disrupt glucose metabolism, but circulating levels of anti- and pro-inflammatory cytokines do not appear to depend on bacterium’s factors, but on the inflammatory response of the host mucosa.

Our results demonstrated that the bacterial density was negatively correlated with IL-10 cytokine plasma levels. The data shown that although the infection is considered local, it results in systemic impairment of the inflammatory response and depends on the density of *H. pylori* colonization. Moreover, a decrease in IL-10 expression in the gastric mucosa was observed by immunohistochemistry in *H. pylori*-infected patients with an increased density of *H. pylori* colonization and an increased degree of both neutrophil activity and chronic inflammation of the gastric mucosa. Vinagre et al. (36) detected low levels of IL-10 in the gastric mucosa of *H. pylori*-infected patients with an increased degree of inflammation and increased neutrophil activity of the chronic gastritis. Could the decrease in IL-10 expression during *H. pylori* infection, as observed in this study, lead to an increase in bacterial colonization and the bacterial density would to increased glycated hemoglobin levels? Or, conversely, could the increase in bacterial density induce decreased expression of IL-10? Could plasmatic IL-10 levels be used as a marker to detect the possible risk of prediabetes in an *H. pylori-*infected patient? In this study, although a correlation between the levels of cytokines in plasma with cytokines in the gastric mucosa was not demonstrated, the expression of the IL-10 cytokine showed a corresponding systemic and local pattern in *H. pylori* infection. This suggests that plasma levels of IL-10 reflect the load of bacterial colonization and the intensity of inflammatory process in the gastric mucosa, which reinforces the possibility of serum IL-10 being used as a risk marker for prediabetes in *H. pylori*-infected patients. Studies indicated the association between the low levels of IL-10 with the high HbA1c levels. For this reason, it is possible to consider the blood levels of IL-10 as one of the predictors of glycemia (37, 38).

In this study, we found no correlation between plasma levels of IL-10 or TNF-α with HbA1c levels. On the other hand, in the gastric mucosa, we observed a positive correlation between IL-10 and TNF-α expression with HbA1c. Wang et al. (39) observed that plasma levels of IL-10 were positively associated with prediabetes or T2DM, in disagreement with other studies that showed that IL-10 improve impaired insulin signaling (40) and prevent pancreatic beta cell destruction (41). However, we observed in our study that *H. pylori*-infected patients with a higher degree of mucosal inflammation and higher bacterial density had increased levels of HbA1c and reduced levels of IL-10 in the plasma and gastric mucosa. Thus, the increase in HbA1c levels would be dependent on inflammatory response degree of the host mucosa, which is dependent of bacterial density. Most studies found that subjects with insulin resistance or T2DM had increased levels of TNF-α (42). It has been reported that chronic exposure of insulin-producing pancreatic cells (β cells) to the pro-inflammatory cytokine TNF-α can inhibit insulin secretion and induce apoptosis of pancreatic β cells (42). The progression from normal glucose tolerance to prediabetes and T2DM is characterized by continuous defects in β cell function (43). In this study, patients infected with *H. pylori* showed a positive correlation between TNF-α expression in the gastric mucosa and HbA1c levels, which suggests that the pro-inflammatory cytokines induced by *H. pylori* infection play an important role in the disorder of glucose metabolism.

Another question to keep in mind is whether eradicating *H. pylori* infection could reduce HbA1c levels in *H. pylori*-infected patients without prediabetes. Data are conflicting regarding the impact of *H. pylori* eradication on glucose metabolism. *H. pylori* eradication improved the mean HbA1c values in non-diabetic patients (44) and in patients with T2DM (45).

We consider *H. pylori* infection to be a risk factor for prediabetes, and we believe this is an additional reason to eradicate *H. pylori* infection in adulthood. Special attention should be given to those patients with higher bacterial loads and higher degrees of inflammation and neutrophil activity of the chronic gastritis. In these patients, the evaluation of HbA1c levels would be of interest.

More studies are necessary to elucidate whether *H. pylori per se* or host-related factors are involved in disturbances of glycated hemoglobin. Detailed analysis of the levels of pro- and anti-inflammatory cytokines in the gastric mucosa and glycated hemoglobin levels in patients infected with *H. pylori* appears to be of relevance for further study.

## Data Availability Statement

The raw data supporting the conclusions of this article will be made available by the authors, without undue reservation.

## Ethics Statement

The National Committee for Research Ethics (CONEP) approved this study according to CAAEE 07989412.0.0000.5086. The patients/participants provided their written informed consent to participate in this study.

## Author Contributions

SM, NS, and FN conceived and designed the experiments. SM, DNC, DMC, JN, BM, LDS, and MB performed the experiments. SM, JS, DMC, LAS, JN, RG, and FN analyzed the data. NS, RG, and FN contributed reagents, materials, analysis tools. SM, JN, LAS, RG, NS, and FN wrote the manuscript. All authors contributed to the article and approved the submitted version.

## Conflict of Interest

The authors declare that the research was conducted in the absence of any commercial or financial relationships that could be construed as a potential conflict of interest.

## Supplementary Material

FIGURE S1 Association between *H. pylori* infection (HP) with glycated hemoglobin (HbA1c) levels in patients without (Control) or with *H. pylori* infection (HP) according endoscopic diagnosis. Endoscopic diagnosis were evaluated according endoscopic classification of the Sydney System of patients infected with *H. pylori* and with pangastritis (HP gastritis, *n* = 34) or non-infected with peptic ulcer (Control peptic ulcer, *n* = 10).

FIGURE S2 Correlation between cytokines IL-10 and TNF-α, in plasma and in gastric mucosa with degree bacterial density of the gastric mucosa. Cytokines concentration in plasma were measure by CBA, expression of cytokines in the gastric mucosa were evaluated by immunohistochemistry and degree bacterial density was evaluated by Sydney System histological classification. Correlation between bacterial density of the gastric mucosa with IL-10 **(A)** and TNF-α **(B)** plasma concentration of 47 patients. Correlation between bacterial density of the gastric mucosa with expression of IL-10 **(C)** and TNF-α **(D)** in gastric mucosa of 37 patients. Correlations were determined by Spearman’s correlation tests. Level of significance, *p* < 0.05.

FIGURE S3 Association between *H. pylori* infection (HP) with glycated hemoglobin (HbA1c) levels in patients non-prediabetic (Non-prediabetic HP) and prediabetic (Prediabetic HP) with *H. pylori* infection, patients non-prediabetic (Non-prediabetic control) and prediabetic (Prediabetic control) without *H. pylori* infection. HbA1c levels were evaluated in the plasma of patients Non-prediabetic HP (*n* = 22), Prediabetic HP (*n* = 40), Non-prediabetic control (*n* = 13) and Prediabetic control (*n* = 9) using a method certified by the National Glycohemoglobin Standardization Program (NGSP). *p*-value was determined by unpaired two-tailed *t*-test. **p* < 0.05 compared Non-prediabetic HP and Non-prediabetic control group. ***p* < 0.0001 compared Non-prediabetic control and Prediabetic control groups. ^#^*p* < 0.0001 compared Non-prediabetic HP and Prediabetic HP groups. Level of significance, *p* < 0.05.
